# Review of "Advances in Silicon Carbide Processing and Applications" 1^st ^Edition by Steven E. Saddow and Anant Agarwal

**DOI:** 10.1186/1475-925X-4-33

**Published:** 2005-05-17

**Authors:** Alton Horsfall

**Affiliations:** 1 School of Electrical, electronic and Computer Engineering, University of Newcastle, Newcastle, Tyne and Wear, NE1 7RU, UK

"Advances in Silicon Carbide Processing and Applications" specifically targets the technology of two key application areas, propulsion systems in electronic vehicles and sensors for deployment in extreme environments. Edited by Steven Saddow & Anant Agarwal, two highly respected researchers in the field of SiC, with contributions from a range of eminent scientists, the book offers a real insight to these fascinating areas. The technology lead approach on these two areas sets the book apart from other recent offerings, such as 'Process Technology for SiC Devices', by Zetterling [1] and 'Silicon Carbide, Recent Major Advances', by Choyke, Matsunami and Pensl [2].

After the introductory chapter one the growth and potential market of silicon carbide wafers, is suitable for those people who are new to the field of SiC and offers a strong background to support the remainder of the material presented in the later chapters. The indication that the market forces for SiC power electronics will be in the 300V plus market however, goes against the significant inroads made by both Infineon [3] and Cree [4] into the switch mode power supply market for domestic customers. This is followed by a chapter which focuses on the technological requirements of implantation and annealing for the production of the selectively doped regions required for device fabrication. With the crystal lattice of SiC being particularly dense, the damage caused by ion implantation is severe and significantly degrades the carrier mobilities in these regions. The chapter provides a thorough review of the techniques being used to investigate the extent of this damage and the measures being used to minimise it in device structures.

The chapters at the rear of the book focus on the development of power transistors (both MOSFETS and bipolars) and their suitability for replacing silicon based power devices in power electronic circuitry, as would be suitable for propulsion systems. Because SiC devices offer the possibility of operation with junction temperatures substantially above the 175°C limit associated with Si power devices, they can be operated without the need of heavy and cumbersome heat sinks. This, along with their higher efficiency makes them particularly suitable for mobile propulsion systems. The material properties of SiC also make it suitable for the fabrication of high power radio frequency devices, such as IMPATT diodes and bipolar transistors and the final section shows the recent research in this area which offers a potentially lucrative market for SiC devices as they offer superior properties in comparison to silicon.

The remaining chapter focuses on the development sensor structures for deployment in environments beyond those possible with current technology, such as the detection of gas species in exhaust systems for both transportation and domestic systems. The chapter offers a review of the latest results from research groups in both Sweden and the US, who are arguable at the forefront of research in this field. This includes an introduction to the operation of the devices and the importance of the dissociation of gas species with a catalytic metal, before demonstrating the influence of a range of gas species (such as hydrogen, nitrous oxides and ammonia) to the behaviour of these devices. Results in the chapter illustrate the response of these sensors to gas species whilst being deployed in situ, such as a car exhaust, where a mis-firing cylinder can be easily identified from the change in gas mixture in the manifold and give details about the practical nature of deploying these sensors, such as mounting the SiC device and electrical connections. The application of this technology to biomedical applications is an area ripe for development, as silicon carbide material properties are far superior to those observed in traditional semiconductors, such as silicon. To date only a single reference exists showing development in this field [5]. The content of this chapter alone is sufficient to warrant the purchase of this book and given the popularity of this volume with our postgraduate students, I would recommend it to anyone wishing to gain more knowledge in this exciting field of work.
